# Black Pastors as Change Agents Promoting COVID-19 Vaccination in South Carolina: Application of Diffusion of Innovations Theory

**DOI:** 10.12691/ajphr-13-2-6

**Published:** 2025-04-11

**Authors:** DaKysha Moore, Lisa N. Mansfield, Nicole Caviness-Ashe, Elijah O. Onsomu

**Affiliations:** 1 Department of Communication Sciences and Disorders, North Carolina Agricultural and Technical State University, Greensboro, NC; 2 School of Nursing, The University of North Carolina at Chapel Hill, Chapel Hill, NC; 3 Cancer Prevention and Control Training Program, Heersink School of Medicine, The University of Alabama at Birmingham, Birmingham, AL; 4 Division of Nursing, Winston-Salem State University, Winston-Salem, NC

**Keywords:** Church Leaders, Community, COVID-19, Faith Community, Health Communication, Religion, Vaccine

## Abstract

During the COVID-19 pandemic, many minority communities tried different strategies to increase their members’ vaccination rates. This study uses diffusion of innovations theory [1] with a focus on “the sequence of change agent roles” to explore Black pastors’ approach to COVID-19 vaccine hesitancy. Ten semi-structured interviews gauged how Black pastors understood their role in increasing vaccine use in their communities. Seven themes emerged from analysis: creating awareness of change; establishing a connection; seeking to understand; encouraging the new behavior; leveraging the power of interpersonal communication; maintaining support; and providing future assistance. Results im-ply that by continuing to work with healthcare providers, Black pastors can help to in-crease awareness about annual COVID-19 boosters among their church members and in surrounding communities.

## 1. Introduction

In 2019, at the beginning of the COVID-19 pandemic, racial and ethnic minority groups were at greater risk for SARS-CoV-2 infections. Blacks’ risk was the highest [2]. According to the Centers for Disease Control and Prevention [3], Blacks were more likely to be diagnosed with COVID-19; 2.5 times more likely to be hospitalized; and nearly twice as likely to die from COVID-19 compared to Whites. In South Carolina, Black men and the elderly experienced higher rates of death and hospitalization for COVID-19 than did other residents [4]. Blacks were more susceptible to COVID-19 for several reasons, including living in congested areas, not being able to telework, and high rates of chronic conditions [5].

In response to these COVID-19 disparities, national public health efforts prioritized making COVID-19 testing and vaccination more accessible and affordable for high-risk populations [6]. However, rates among Blacks remained low due to mistrust [7,8]. Mansfield et al. [9] found concerns about the COVID-19 vaccine among Blacks living in Los Angeles based on historical medical mistreatment and racism.

In South Carolina, more than 75% of COVID-19 cases, hospitalizations, and deaths were among those who were not fully vaccinated by July 2021, although an estimated half of the population had had at least one dose of the vaccine [10]. As of 2022, the percentage of South Carolinians fully vaccinated increased to 58% [11].

Many newspaper headlines and television news stories across the country described the impact of Black pastors in increasing vaccine uptake among their congregants and local communities. For example, the New York Times reported “‘A Safe Space’: Black Pastors Promote Vaccinations from the Pulpit” p. 1 [12], and U.S. News and World Report ran “To Save Their Communities, Black Ministers Preach the Gospel about the COVID-19 Vaccine” p. 1 [13]. Their recognition of the importance of pastors of Black churches as trusted voices to help increase COVID-19 vaccine rates in the Black community is no surprise. The Black church has long been an effective ally in combating various diseases, suggesting pastors may be change agents in promoting good health [14]. A host of research studies attests its reach to the Black community and promotion of a holistic approach to healthcare [14]. Some studies suggest that faith-based programs can help to increase physical activity among Blacks [15–17] and create more awareness about diseases, such as high blood pressure and diabetes [18–20], to help with weight loss and management [21].

Studies published since the onset of COVID-19 show Black pastors confronting the pandemic. One study found that Blacks who identify with the “Black Protestant tradition” and attend church at least once each month were more likely to be vaccinated for COVID-19; over 50% of survey respondents indicated that their pastors encourage inoculation for the virus [22]. Dada et al. [23] discussed the importance of Black pastors partnering with other trusted members in the Black community, such as Black medical providers, to increase COVID-19 vaccination rates. Furthermore, the National Black Church Initiative (NBCI), a collaboration between Black and Latino churches, partnered with the CDC in 2021 to help increase COVID-19 vaccination rates. These churches became established community vaccination clinics [24].

In 1962, E. M. Rogers first proposed the “diffusion of innovations theory” to better understand how using change agents, or influential and effective sources, to disseminate information encourages the adoption of new ideas or behaviors (Rogers, 2003). “The innovation is an idea, practice, or object perceived as new by an individual or other unit of adoption. The diffusion process typically involves both mass media and interpersonal communication channels” p. 415 [25]. A change agent is part of the interpersonal communication channels, “[a]n individual who influences clients’ innovation-decisions in a direction deemed desirable by a change agency. …Change agents often use opinion leaders in a social system as their lieutenants in diffusion activities” p. 27 [1]. The innovation may be adopted faster if effective communication of the message connects recipients with the overall idea [26]. The “sequence of change agents” describes the different stages of communication that change agents can use to get people to accept the new product or behavior. This paper uses these ideas to elucidate Black pastors’ position as change agents in disseminating information about COVID-19 vaccinations.

More specifically, the theory’s communication domain guides this qualitative analysis and the organization of themes. Pastors are conceived as change agents because they worked with different local, state, and/or health agencies to disseminate information and/or provide opportunities to administer the COVID-19 vaccines at their churches or local community vaccination sites. The study focuses on the “the sequence of change agent roles” described in Rogers p. 369 [1] as it pertains to pastors of predominantly Black churches in South Carolina.

## 2. Methods

This paper is part of a larger study that explored the communication channels pastors of predominantly Black churches in South Carolina used in response to COVID-19 and vaccination [27]. The churches were located in both rural and urban areas. We used a qualitative, phenomenological design that allowed us to understand how pastors worked with their congregations and surrounding communities to increase awareness about COVID-19 vaccines and to promote vaccination [28]. The lead author’s Institutional Review Board (IRB) approved the study.

The interview guide consisted of eleven questions adapted from a study on the communication channels used in the Black church to discuss HIV/AIDS [29]. Here, the questions focused on background information about the churches and dissemination practices the Black pastors used to create awareness of COVID-19 testing and vaccinations. The validity and reliability are addressed through the use of a similar questionnaire that addresses a health topic among a comparable population.

### 2.1. Sample

We used snowball sampling to identify and contact pastors at predominately Black churches in South Carolina. By asking for referrals from each pastor, we found 10 pastors who agreed to participate in the study. The number of participants contacted was less than 15. It was a challenge to speak with pastors who fit the demographics of the study. Because of the pandemic, many churches did not have someone in the office. Data saturation could be reached with sample sizes ranging from six to eight participants [30]. Data saturation was achieved at 10 participants for this study, which is still considered acceptable. There was not a specific rule for deciding on saturation. However, according to Morse [31], when a researcher is conducting “a phenomenological study and interviewing each person many times, one has a large amount of data for each participant and therefore needs fewer participants in the study (perhaps only 6 to10)” (pp. 4–5). For this study, the researchers did not interview the participants more than once, but the interviews were in-depth based on the questions and provided enough information.

Study design used semi-structured interviews, but the set questions allow discussion of new ideas resulting from responses. With the pastors’ approval, each interview was recorded over the telephone during July 2021. The IRB approved the pastors giving consent verbally over the telephone. Most interviews lasted from ten to 30 minutes.

### 2.2. Data Analysis

Interviews were recorded and transcribed using recording software (Otter.ai) and compared and reviewed for accuracy. The 10 transcriptions were uploaded into the soft-ware QDAMiner Lite for coding [32]. They were coded based on “the sequence of change agent roles” p. 369 [1] as part of the diffusion of innovations. The codes were grouped into categories based on similar patterns within the data. The categories were then assessed for similarities and differences in patterns and relationships, and themes identified based on consistencies in the pastors’ communication channels for disseminating COVID-19 vaccine information. Themes were then grouped using the communication sequence steps [1].

## 3. Results

The sample included pastors in South Carolina who represented the African Meth-odist Episcopal (AME) and Baptist denominations. Most were men (70%). Congregation sizes ranged from approximately 100 to thousands of registered members. Their descriptions of their involvement in communicating information about COVID-19 vaccines to their church and local communities show them as change agents. A total of seven themes emerged, which correspond with the sequences of Rogers’s seven roles of a change agent (see Table 1).

### 3.1. Creating Awareness of the Change

Pastors identified their function in helping to educate their church members about, not only COVID-19, but also the importance of becoming knowledgeable about the vaccine. Pastors felt that it was their charge to disseminate COVID-19 vaccine information during church services.

“I just think that the more information we have, … the better off we are as a whole, and the more solid information that we’re able to provide people, I think, the more we’re able to clear up some of the misconceptions … spread about the vaccine.” (**Pastor 3, Baptist**)

Some pastors made the decision to receive the vaccine in front of their congregations to emphasize its importance. Some pastors shared their vaccine experiences with their church members. They indicated that showing images of themselves taking the vaccine demonstrate they were not only encouraging others, but also willing to take the lead.

### 3.2. Connecting

Pastors described having an established relationship with church members and the surrounding community. They built on that trust to address reasons for hesitancy and other factors to consider when deciding to get vaccinated.

“I think church plays a very pivotal role in the distribution of information, and the vaccine itself; I do believe the African American church is a vital role in the lives of African Americans. And there’s a certain trust factor there that comes along with the church and the Minister. And I do believe the more it is promoted from the church’s standpoint, I believe the more we see it will eventually become because of that level of trust of the church in the African American community.” (**Pastor 3, Baptist**)

### 3.3. Seeking to Understand

Pastors did not try to force members to get vaccinated but reported using a technique of empathetic listening to understand any reluctance.

“Since you are a part of the community, you have to encourage people to be vaccinated. I admit that is a tight rope to walk because there are all types of beliefs in the church. There are political beliefs, health beliefs, and there are some people who love the Lord, but they truly believe in holistic medicine and health medicine.” (**Pastor 10, Baptist**)

“We’ve had conversations virtually relative to the vaccines and the side effects, having open discussions, you know, with individuals having concerns or even people who had different types of results—meaning side effects or whatever the case is—and how long it took for them to get over certain things, because everybody is different.” (**Pastor 7, AME**)

“There’s a lot of different misinformation floating around, so I always try to encourage people to consult with your physician and then you make the best-informed decision regarding the vaccine based on your discussion with your physician.” (**Pastor 3, Baptist**)

### 3.4. Encouraging New Behavior

Pastors used their influence to encourage those who were hesitant by endorsing COVID-19 vaccines. They presented the vaccines as the best way to protect themselves and their families.

“Well, I personally endorsed the vaccination in congregations—still do. I have received a vaccination myself, and I encouraged my congregation to get vaccinated as well.” (**Pastor 4, Baptist**)

“And so I take it very personal, very personal to me, and I probably reach the point of irritation at times—that’s how much I talk about ‘be safe, be careful’— you know—’get vaccinated’. We do have a part of the congregation, especially the younger parts of our congregation, that just don’t want to get the vaccine. And then we have, you know, the older … sectors that do not want to get it either. So, I’m constantly trying to, you know, give information and explain how dangerous it is, and they should still wear their mask, and we provided masks and supplies to people.” (**Pastor 2, AME**)

“Our older generation, they have done great. They are going and receiving the vaccination. Now, you do have some, because of health issues, who have not been going to get it. That is one of the factors, such as diabetes, kidneys, heart congestion, kidney problems, and like that, but they have been the front runners.” (**Pastor 8, Baptist**)

### 3.5. Leveraging the Power of Interpersonal Communication

Pastors acknowledged the importance of tone and use of language when engaging with members about getting vaccinated.

“And again, we do have some members we know that are skeptical—actually, a member called, and she said, ‘Pastor, I need encouragement.’ I said okay. We talked for a while, it’s like I made her laugh for a while about the man who was in the line and scared to take the vaccine and was told take the vaccine or die, and he says, ‘I will take the vaccine.’ She said she had butterflies and all kinds of jittery things going on, but she took it.” (**Pastor 6, AME**)

“I find it vitally important because, as we know, disseminating information from our standpoint is crucial to an African American community. So, I think it’s vitally important that we submit all information that we possibly can at any given form.” (**Pastor 4, Baptist**)

“We’ve been keeping our people informed about the lows and highs of the cases. We have a COVID ministry. We designed that when this happens..., one to make sure that we follow the CDC guidelines, along with the guidelines for our church, to make sure that our people are safe and protected.” (**Pastor 8, Baptist**)

### 3.6. Maintaining Support for COVID-19 Vaccines

The pastors described using different forms of interpersonal and mass communication channels to encourage more people in the church community to become immunized and continue getting the recommended vaccinations for COVID-19.

“What I did, I posted some things, personally, especially when it first came out. Let’s share some things on social media. On my personal page, not on the church’s page. But we did have our Bishop offer the vaccine at different locations in the church, and we did share that information [via] social media, text message, email. We shared those things. And ironically, we have an opportunity, we’re partnering with the ... community resource center, partnered with them to start a decent PSA to get the word out and have some people influence and try to make those appeals so that people will get vaccinated.” (**Pastor 2, AME**)

“I do weekly pastoral remarks, announcements of emphases each week, something related to vaccinations.” (**Pastor 1, Baptist**)

Pastors also described using different media channels, such as social media platforms, to increase awareness about COVID-19 vaccination and to deliver messages about the importance of getting vaccinated to their congregations.

“We have our Facebook page, so we post things there. And we post whenever we’ve had the vaccinations, and we have post updates about COVID. And we just keep posting on Facebook. And I’m a text person; I text my officers at least twice a week. And there’s always something uplifting and then, stay safe.” (**Pastor 6, AME**)

### 3.7. Assisting in the Future

The pastors stated that during the pandemic, they were willing to continue the relationship with those in the community who could help to provide COVID-19 vaccination for their congregations. One pastor discussed making sure to help people get to vaccination clinics through the religious affiliation: “*Within the church district, we have had several locations where people could go get vaccinated—so at our larger churches with large parking lots—large areas where people could go” (****Pastor 9, AME****)*. Some pastors not only provided information about COVID-19 vaccine but took people to clinics and/or established vaccine sites at their churches.

## 4. Discussion

Using diffusion of innovations theory, we highlight the importance of Black pastors’ position as change agents for promoting COVID-19 vaccination in Black communities in South Carolina. According to Rogers [1], change agents can help to pass information from “agency” to “audience.” In working with state and local health agencies, pastors play a vital role in disseminating information about COVID-19 vaccines to their congregations and the surrounding areas. They used different strategies, such as specific word choice, to avoid a disparaging tone and to learn why some church members were hesitant to take a COVID-19 vaccine. At times, they were challenged to provide too much information, which can cause misunderstandings about an innovation [1]. Caron and Dorsey [33] de-scribe the importance of addressing “competing messages” about the COVID-19 vaccine and encouraging more messages among clergy. Pastors in this study sought to empathize with their church members’ concerns [34], while sharing facts from reputable sources to convey the benefits of vaccination. Their trusting relationship is vital. Pastors emphasized the importance of not only disseminating accurate, health-based information about the recommended vaccines, but providing access, especially during the early months of distribution. Pastors set up clinics at their churches, provided access to clinics, and/or disseminated information about clinics. Abdul-Mutakabbir et al. [35] describe an effective strategy relying on Black pastors and medical providers to increase awareness and remove structural barriers to accessing vaccines, such as deploying mobile clinics. These strategies were crucial for reducing inequitable access to COVID-19 vaccines during the initial distribution.

Disparities in vaccine access and use between Blacks and non-Hispanic Whites narrowed throughout the pandemic [36]. Reasons for the decline could be attributed to 1) use of trusted messengers to promote COVID-19 vaccination in communities with a history of medical mistrust and mistreatment; 2) improved understanding of the inequities contributing to racially and ethnically marginalized groups’ high risk for COVID-19; and 3) increased access to vaccination clinics [36]. Black pastors have served as change agents, increasing awareness and reducing barriers by taking people to vaccination clinics or enabling vaccination at their churches.

Previous health programs have used pastors as change agents. Most pastors have an established relationship with their church members and the surrounding community, and their messaging may be more culturally centered than messaging from unconnected members of the healthcare system [14,37]. Historical medical mistrust and contemporary experiences of discrimination in healthcare have been cited as reasons why Blacks are apprehensive participating in medical studies [38]. Their pastors understand why church and community members may be reluctant to take the vaccine. Partnering with trusted Black pastors is imperative in educating Black communities about COVID-19 vaccination and other healthcare innovations. Future healthcare messaging should be culturally tailored to Black communities and address their specific concerns.

This study has several limitations. First, findings may not apply to Black pastors in other states. Second, the 10 pastors are clustered in two areas, mainly the coast and up-state; Black pastors in the central and western regions of South Carolina may consider different strategies to help increase COVID-19 vaccine uptake among their congregants. Third, the Black pastors represent only two denominations. Finally, the researchers did not interview church members to understand their attitudes about the pastors’ COVID-19 vaccination messages.

## 5. Conclusions

This study used the diffusion of innovations theory to identify the function of pastors in predominantly Black churches as change agents in disseminating COVID-19 vaccine information in South Carolina. It highlights the importance of developing strategies with trusted messengers, such as Black pastors, to improve communication and health awareness in communities that have endured discrimination for centuries. Pastors who disseminate COVID-19 vaccine information continue to help protect the health of their communities.

## Figures and Tables

**Table 1.** Themes corresponding with the “Seven Roles of a Change Agent”

Theme	Definition of Diffusion of Innovations Step Communication Sequence (Rogers, 2003, pp. 369–370)
Creating awareness of the change	To develop a need for change
Connecting	To establish an information exchange relationship
Seeking to understand	To diagnose problems
Encouraging the new behavior	To create an intent to change in the client
Leveraging the power of interpersonal communication	To translate an intent into action
Maintaining support for vaccine	To stabilize adoption and prevent discontinuance
Assisting in the future	To achieve a terminal relationship
